# Supplementary material for: Temporal profiles of avalanches on networks
Source: Nat Commun. 2017 Oct 31;8:1227. doi: 10.1038/s41467-017-01212-0 (PMC5663919; doi:10.1038/s41467-017-01212-0)
Supplement: Supplementary file 1 — Supplementary Information [file 41467_2017_1212_MOESM1_ESM.pdf]

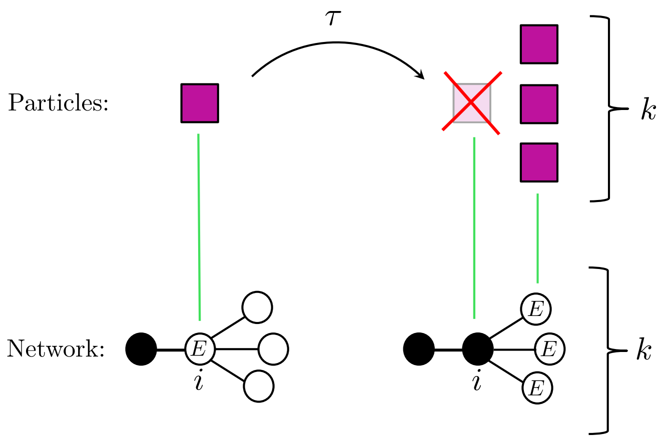


Supplementary Figure 1: | **Linking branching processes to cascades on networks.** The green lines demonstrate the analogy between the particles of a continuous-time branching process (top), and the exposed vulnerable (EV) nodes of a cascade process on an undirected network (those nodes marked "E" in the bottom diagrams), as described in Supplementary Note 1. Black nodes are active and white nodes are inactive; note that only vulnerable nodes are shown here (so the actual degree of node $i$ is at least $k+1$).

# Supplementary Note 1: Linking branching processes to discrete-state dynamics on networks

In this Supplementary Note we demonstrate how branching processes can be used to describe unidirectional dynamics on configuration-model networks. The paradigm of continuous-time branching processes describes particles who each live for a lifetime $\tau$, where the value of $\tau$ is drawn from a prescribed distribution. In the Markov case, the distribution of $\tau$ values is exponential (and we choose the timescales so that the mean of the distribution is 1); in the Galton-Watson process time is discrete, so every particle has a lifetime of exactly 1 time unit. (The framework we describe here can also be used to link non-Markovian dynamics with the appropriate (Bellman-Harris) branching processes [1]; however, in this paper we restrict our attention to the Markovian continuous-time, and discrete-time, cases). At the end of its lifetime, the particle dies; simultaneously, a number $k$ of identical "children" particles appear, where $k$ is a random integer from a distribution $q_k$ (the offspring distribution). Each avalanche of the branching process is initiated at time 0 by a single particle, and terminates at time $T$ when there are no particles remaining alive; the temporal evolution of the cascade process (i.e., the avalanche shape) can be described in terms of the number of particles alive at time $t$, for times $t$ with $0 < t < T$.

Supplementary Figure 1 shows how unidirectional dynamics on a network can be described in terms of a branching process. First, we define a node as "exposed" if it is currently inactive but one of its neighbouring nodes is active. We identify a particle of the branching process with an exposed vulnerable (EV) node of the network, i.e., a node that is currently inactive, but is linked to an active node, and that will eventually become active itself. An EV node (such as the node $i$ in Supplementary Figure 1) remains in the EV state for a time $\tau$ until it becomes active. Since the node $i$ is then no longer an EV node, it can be considered to be a "dead" particle of the branching process. However, by becoming active, node $i$ has also simultaneously exposed all of its inactive

neighbours, and if $k$ of these are vulnerable (where $k$ is less than or equal to the degree of node $i$) then $k$ new EV nodes (particles) have been created. The number $k$ of these "children particles" depends on the degree of node $i$ and on the probability $r = \sum \hat{q}_k$ that a node reached along a random (in-) edge is vulnerable. An EV node has $k'$ inactive neighbours with probability $\hat{q}_{k'}/r$, and since each inactive neighbour is (independently) vulnerable with probability $r$, the offspring distribution (the probability that a particle generates $k$ children particles (EV nodes) when it dies) is given by

$$q_k = \frac{1}{r} \sum_{k'=k}^{\infty} \hat{q}_{k'} \left( \begin{array}{c} k' \\ k \end{array} \right) r^k (1-r)^{k'-k}. \tag{1}$$

The corresponding generating function is given by equation (5) of the main text.

By differentiating equation (5) of the main text, it is straightforward to verify the simple relations

$$f'(1) = \sum_k k\, q_k = \sum_k k\, \hat{q}_k \tag{2}$$

and

$$f''(1) = \sum_k k(k-1) q_k = r \sum_k k(k-1) \hat{q}_k. \tag{3}$$

In particular, $f''(1)$ is infinite if $\hat{q}_k \sim C\, k^{-\gamma}$ as $k \to \infty$ for values of the power-law exponent $\gamma$ between 2 and 3. By considering the asymptotic expansion of $f(x)$ near $x = 1$, as in Supplementary Equation (46) of Supplementary Note 6, it can be seen that if $\hat{q}_k \sim C\, k^{-\gamma}$ then the offspring distribution $q_k$ also has a power-law tail, with the same exponent $\gamma$.

## Supplementary Note 2: Derivation of average avalanche shape

In our theoretical approach, we model the avalanche dynamics using a continuous-time branching process [1]. Each "particle" survives for a random lifetime $\tau$ (drawn from the exponential distribution with mean 1) and at the end of its lifetime, is replaced by $k$ "children", where the number of offspring $k$ is $0, 1, 2, \ldots$ with probability $q_k$. The mean number $\xi$ of children per parent is given by equation (3) of the main text; the critical case $\xi = 1$ is of particular interest, but our derivation is also valid for other cases.

Following the notation of Chapter III of [1], we define $Z(t)$ as a one-dimensional continuous time Markov branching process representing the number of alive particles at time $t$. The (time-homogeneous) transition probabilities are defined as

$$P_{1j}(t) = \text{Prob}\left\{ Z(\tau + t) = j | Z(\tau) = 1 \right\} \tag{4}$$

and the corresponding generating function is

$$F(s,t) = \sum_k P_{1k}(t) s^k. \tag{5}$$

Note that the probability of extinction by time $t$ is

$$Q(t) \equiv \text{Prob}\left\{ Z(t) = 0 | Z(0) = 1 \right\} = F(0,t). \tag{6}$$

We consider an avalanche as a process starting at time $t = 0$ with a single particle ($Z(0) = 1$), with lifetimes of particles that are exponentially distributed with parameter 1. Upon death, a

particle leaves $k$ offspring with probability $q_k$, $k = 0, 1, 2, \ldots$; the generating function for the offspring distribution is defined in equation (5) of the main text, with $\xi = f'(1)$.

For a fixed duration $T > 0$, we define the avalanche path probability $\pi_n(t)$ to be the probability that $n$ particles are alive at time $t$ ($0 \leq t \leq T$), conditioned on there being exactly one particle alive at time 0 and exactly one particle alive at time $T$:

$$\pi_n(t) = \text{Prob}\left\{Z(t) = n | Z(0) = 1 \text{ and } Z(T) = 1\right\}. \tag{7}$$

Because of the Markov property, $\pi_n(t)$ is proportional to the product of the probabilities of having a path that (i) has $n$ particles alive at time $t$ and (ii) goes from $n$ particles at time $t$ to one particle at time $T$, so

$$\pi_n(t) \propto P_{1n}(t) P_{n1}(T - t). \tag{8}$$

We can write

$$P_{n1}(T - t) = n \left[P_{10}(T - t)\right]^{n-1} P_{11}(T - t) \tag{9}$$

to reflect the fact that the $n$ particles active at time $t$ can be reduced to a single particle at time $T$ only if $n - 1$ of them have no descendants alive at time $T$, with the remaining one (which can be chosen in $n$ ways) having one descendant at time $T$. The correctly normalized avalanche path probability distribution is therefore

$$\pi_n(t) = \frac{P_{1n}(t)\, n \left[P_{10}(T - t)\right]^{n-1} P_{11}(T - t)}{\sum_n P_{1n}(t)\, n \left[P_{10}(T - t)\right]^{n-1} P_{11}(T - t)}. \tag{10}$$

The denominator of Supplementary Equation (10) can be written as

$$F'\left(P_{10}(T - t), t\right) P_{11}(T - t), \tag{11}$$

where, for brevity, the prime denotes differentiation with respect to the generating function variable $s$ (i.e., the first argument of $F(s, t)$). Noting that $P_{10}(t) = F(0, t) = Q(t)$ and $P_{11}(t) = F'(0, t)$, the denominator of Supplementary Equation (10) can be further simplified to

$$F'\left(F(0, T - t), t\right) F'(0, T - t) = F'(0, T), \tag{12}$$

where we have used the Markov property [1]

$$F(s, T) = F\left(F(s, T - t), t\right). \tag{13}$$

Thus, the avalanche path probability distribution is

$$\pi_n(t) = \frac{P_{1n}(t)\, n \left[P_{10}(T - t)\right]^{n-1} P_{11}(T - t)}{F'(0, T)}, \tag{14}$$

and the generating function for this distribution is

$$\begin{aligned} G(s, t) &= \sum_n \pi_n(t) s^n \\ &= \frac{1}{F'(0, T)} \sum_n P_{1n}(t)\, n \left[P_{10}(T - t)\right]^{n-1} P_{11}(T - t) s^n \\ &= \frac{s F'\left(s P_{10}(T - t), t\right) P_{11}(T - t)}{F'(0, T)}. \end{aligned} \tag{15}$$

The average avalanche shape is determined by the expected number of particles alive at time $t$, where the expectation is over the set of avalanche paths conditioned as in Supplementary Equation (7). We calculate this using the generating function $G(s,t)$ as follows:

$$G'(1,t) = \frac{1}{F'(0,T)}\left[F'\left(P_{10}(T-t),t\right)P_{11}(T-t) + F''\left(P_{10}(T-t),t\right)P_{10}(T-t)P_{11}(T-t)\right]$$
$$= 1 + \frac{F(0,T-t)F'(0,T-t)F''\left(F(0,T-t),t\right)}{F'(0,T)}. \quad (16)$$

Since we are considering Markov branching processes, we can use the Kolmogorov forward and backward equations [1] for the generating function $F(s,t)$ :

$$\frac{\partial}{\partial t}F(s,t) = \left(f(s)-s\right)F'(s,t)$$
$$\frac{\partial}{\partial t}F(s,t) = f\left(F(s,t)\right) - F(s,t), \quad (17)$$

and by eliminating $\partial F/\partial t$ from this pair of equations we write $F'(s,t)$ in terms of $F(s,t)$:

$$F'(s,t) = \frac{f\left(F(s,t)\right)-F(s,t)}{f(s)-s} \qquad \text{for } s \neq 1. \quad (18)$$

Differentiating with respect to $s$ and substituting $F'$ using Supplementary Equation (18) yields

$$F''(s,t) = \frac{\left[f\left(F(s,t)\right)-F(s,t)\right]\left[f'\left(F(s,t)\right)-f'(s)\right]}{\left[f(s)-s\right]^2}, \quad (19)$$

and evaluating at $s = F(0,T-t)$ and using Supplementary Equation (13), we have

$$F''\left(F(0,T-t),t\right) = \frac{\left[f\left(F(0,T)\right)-F(0,T)\right]\left[f'\left(F(0,T)\right)-f'\left(F(0,T-t)\right)\right]}{\left[f\left(F(0,T-t)\right)-F(0,T-t)\right]^2}. \quad (20)$$

Using Supplementary Equations (18) and (20) in Supplementary Equation (16) gives

$$G'(1,t) = 1 + \frac{F(0,T-t)\left[f'\left(F(0,T)\right)-f'\left(F(0,T-t)\right)\right]}{f\left(F(0,T-t)\right)-F(0,T-t)}$$
$$= 1 + \frac{Q(T-t)\left[f'\left(Q(T)\right)-f'\left(Q(T-t)\right)\right]}{f\left(Q(T-t)\right)-Q(T-t)}, \quad (21)$$

where $Q(t) = F(0,t)$ solves the ordinary differential equation obtained from the second of Supplementary Equations (17) at $s = 0$:

$$\frac{dQ}{dt} = f(Q) - Q, \qquad \text{with } Q(0) = 0. \quad (22)$$

We have therefore reduced the problem of calculating the average avalanche shape to the solution of a single ordinary differential equation. Given an offspring distribution and a duration $T$, we can easily solve Supplementary Equation (22) using standard numerical methods for $t = 0$ to $T$, then substitute $Q(t)$ into Supplementary Equation (21) to obtain the average avalanche shape

$$A(t) \equiv G'(1,t) - 1, \quad (23)$$

as given in equation (4) of the main text.

To calculate the variance of the avalanche shape, we again use the generating function $G(s,t)$ to write

$$\mathrm{var}\,(n(t)) = \langle n^2 \rangle - \langle n \rangle^2 = G''(1,t) + G'(1,t) - \big(G'(1,t)\big)^2 . \tag{24}$$

Differentiating Supplementary Equation (15) with respect to $s$, then setting $s$ equal to 1 (and using the identities $P_{10}(T-t) \equiv F(0,T-t)$ and $P_{11}(T-t) = F'(0,T-t)$) yields

$$G''(1,t) = \frac{F'(0,T-t)F(0,T-t)}{F'(0,T)} \left\{ 2F''\left(F(0,T-t),t\right) + F'''\left(F(0,T-t),t\right)F(0,T-t) \right\} . \tag{25}$$

Using Supplementary Equation (18) on the first term, and Supplementary Equation (19) and differentiation of Supplementary Equation (18) on the terms in braces leads (after some algebra) to the result

$$\begin{aligned}
\mathrm{var}\,(n(t)) =& \frac{Q(T-t)}{\left[f(Q(T-t) - Q(T-t)\right]^2} \times \\
& \times \left\{ f(Q(T-t)) \left[ f'(Q(T) - f'(Q(T-t) - Q(T-t) f''(Q(T-t) \right] \right. \\
& \quad + Q(T-t) \left[ f'(Q(T-t))^2 - f'(Q(T)) f'(Q(T-t)) \right. \\
& \quad \left. \left. + f''(Q(T)) \left[ f(Q(T)) - Q(T) \right] + Q(T-t) f''\left(Q(T-t)\right) \right] \right\} .
\end{aligned} \tag{26}$$

## Supplementary Note 3: Exactly solvable case

We consider binary fission, with $q_0 = \frac{1+\mu}{2}$ and $q_2 = \frac{1-\mu}{2}$ being the only non-zero offspring probabilities, where $\mu < 1$ measures the deviation from criticality (the branching number is $\xi = 1 - \mu$). The generating function for the offspring distribution is

$$f(s) = \sum_k q_k \, s^k = \frac{1+\mu}{2} + \frac{1-\mu}{2} s^2, \tag{27}$$

and the ordinary differential equation of Supplementary Equation (22) can be solved exactly for $Q(t)$ to yield

$$Q(t) = \frac{(1+\mu)\left(1 - e^{-\mu t}\right)}{1 + \mu - (1-\mu)e^{-\mu t}}. \tag{28}$$

Using these functions in Supplementary Equation (21) gives the exact avalanche shape

$$A(t) = G'(1,t) - 1 = \frac{(1-\mu^2)\left(1 - e^{-\mu t}\right)\left(1 - e^{-\mu(T-t)}\right)}{\mu\left(1 + \mu - (1-\mu)e^{-\mu T}\right)}, \tag{29}$$

which is clearly invariant under interchange of $t$ and $T-t$ at a fixed value of $T$. Hence the average avalanche shape is symmetric about the point $t = T/2$ for all values of $\mu$ (cf. Fig. 2 of the main text).

In the critical limit $\mu \to 0$, Supplementary Equation (28) simplifies to

$$Q(t) = \frac{t}{2+t} \tag{30}$$

and the average avalanche shape is a parabolic function of $t$ with maximum at $t = T/2$:

$$A(t) = \frac{t}{2+T}(T-t) = \frac{T^2}{2+T} \frac{t}{T} \left(1 - \frac{t}{T}\right), \tag{31}$$

The corresponding variance of the avalanche shape is found from Supplementary Equation (26) to be

$$\frac{T^4}{2(2+T)^2}\left(\frac{t}{T}\right)^2\left(1-\frac{t}{T}\right)^2, \tag{32}$$

and so the coefficient of variation for this critical case is $\mathrm{CV} = 1/\sqrt{2}$, independent of $t$ and $T$.

# Supplementary Note 4: Derivation of non-terminating avalanche shape

We consider here the average shape of all avalanches that have not terminated by the observation time $T$. We use the notation introduced in Supplementary Note 2, but here we define (for a fixed observation time $T > 0$) the avalanche path probability $\tilde{\pi}_n(t)$ to be the probability that $n$ particles are alive at time $t$ $(0 \le t \le T)$, conditioned on there being exactly one particle alive at time 0 and at least one particle alive at time $T$:

$$\tilde{\pi}_n(t) = \text{Prob}\left\{Z(t) = n | Z(0) = 1 \text{ and } Z(T) > 0\right\}. \tag{33}$$

Using the Markov property, we have

$$\tilde{\pi}_n(t) \propto P_{1n}(t)\left[1 - P_{n0}(T-t)\right] = P_{1n}(t)\left[1 - P_{10}(T-t)^n\right]. \tag{34}$$

The correctly normalized avalanche path probability distribution is therefore

$$\begin{aligned}\tilde{\pi}_n(t) &= \frac{P_{1n}(t)\ \left[1 - P_{10}(T-t)^n\right]}{\sum_n P_{1n}(t)\ \left[1 - P_{10}(T-t)^n\right]}\\ &= \frac{P_{1n}(t)\ \left[1 - P_{10}(T-t)^n\right]}{1 - Q(T)},\end{aligned} \tag{35}$$

where we have used the Markov property of Supplementary Equation (13) in the denominator to write

$$\sum_m P_{1m}(t)P_{10}(T-t)^m = F\left(P_{10}(T-t), t\right) = F\left(F(0, T-t), t\right) = F(0, T) = Q(T). \tag{36}$$

The average non-terminating avalanche shape is then given by

$$\begin{aligned}A_{NT}(t) &= \sum_n n\tilde{\pi}_n(t)\\ &= \sum_n \frac{n\, P_{1n}(t)\ \left[1 - Q(T-t)^n\right]}{1 - Q(T)}\\ &= \frac{F'(1,t) - Q(T-t)F'\left(Q(T-t), t\right)}{1 - Q(T)}.\end{aligned} \tag{37}$$

Using the relation given by Supplementary Equation (18) for the second term in the numerator we get

$$A_{NT}(t) = \frac{F'(1,t) - Q(T-t)\frac{f(Q(T)) - Q(T)}{f(Q(T-t)) - Q(T-t)}}{1 - Q(T)}, \tag{38}$$

so it remains only to find an expression for $F'(1,t)$.

Recall that $F'(1,t) = \sum_n nP_{1n}(t)$, which is the expected number of particles alive at time $t$. This quantity therefore measures the average number of events observed at time $t$ across an ensemble comprised of all avalanches (with no conditioning on avalanche duration); as noted in the main text, it provides a simple measure of whether an observed ensemble of avalanches is from a subcritical, critical, or supercritical system. Differentiating the backward Kolmogorov equation (the second of Supplementary Equations (17)) with respect to $s$ and setting $s$ equal to 1 gives the ordinary differential equation satisfied by $F'(1,t)$:

$$\frac{d}{dt}F'(1,t) = \left[f'(1) - 1\right] F'(1,t), \tag{39}$$

which has solution

$$F'(1,t) = \exp\left[(\xi - 1)t\right] \tag{40}$$

(recall $\xi = f'(1)$ is the branching number). Therefore, critical systems (those with $\xi = 1$) have a constant expected number of (unconditioned) events per unit time, while in sub- (respectively, super-) critical systems, the number of events decays (resp., grows) exponentially with the age $t$ of the avalanches.

Substituting from Supplementary Equation (40) into Supplementary Equation (38) gives a formula for the average non-terminating avalanche shape in terms of the extinction fraction $Q(t)$; the asymptotic analysis of this shape is given in Supplementary Note 6.

## Supplementary Note 5: Discrete-time branching processes

Here we consider discrete-time (Galton-Watson) branching processes instead of the continuous-time process used in Supplementary Notes 2–4. The arguments leading to Supplementary Equation (16) remain valid in the discrete-time case, but we cannot now use the Kolmogorov equations to further simplify Supplementary Equation (16). However, the Galton-Watson process has the governing equation [1]

$$F(s, t+1) = f\left(F(s,t)\right), \tag{41}$$

from which we can derive the relations

$$F'(s, t+1) = f'\left(F(s,t)\right) F'(s,t) \tag{42}$$

and

$$F''(s, t+1) = f'\left(F(s,t)\right) F''(s,t) + f''\left(F(s,t)\right) \left[F'(s,t)\right]^2, \tag{43}$$

with initial conditions

$$F(s,0) = s, \quad F'(s,0) = 1, \quad F''(s,0) = 0. \tag{44}$$

For any chosen values of the variable $s$ and duration $T$, we can iterate Supplementary Equations (41)–(43) to find the values of $F(s,t)$, $F'(s,t)$ and $F''(s,t)$ at any (integer) time value $t$ between 0 and $T$; evaluating at the appropriate value of $s$ then enables us to calculate the average avalanche shape $A(t) = G'(1,t) - 1$ from Supplementary Equation (16).

Supplementary Figure 2 demonstrates that the discrete-time and continuous-time (from equation (4) of the main text) formulations give very similar results for the average avalanche shapes; accordingly, we concentrate in the main text on the continuous-time case, for which analytical insights (e.g., asymptotic behaviour) are easier to obtain.

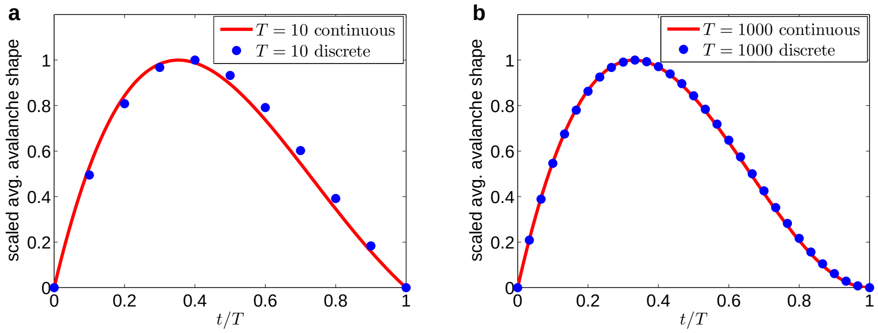


Supplementary Figure 2: | **Comparing discrete-time and continuous-time results.** Average avalanche shapes at criticality ($\xi = 1$) from the continuous-time approach of equation (4) of the main text (curves) and the discrete-time approach of Supplementary Equations (41)–(43) (symbols). The offspring distribution $q_k$ is power-law, with exponent $\gamma = 2.5$. The results of both approaches are very similar even for relatively short-duration avalanches ($T = 10$ in panel (a)), and are almost indistinguishable for long-lived avalanches ($T = 1000$ in panel (b)).

## Supplementary Note 6: Large-$T$ asymptotics at criticality

To consider the asymptotic behaviour of the avalanche shape for a general offspring distribution, we begin with the large-time asymptotics of $Q(t)$. Supplementary Equation (22) can be rewritten as

$$\int \frac{dQ}{f(Q) - Q} = \int dt, \tag{45}$$

and the large-$t$ asymptotic behaviour corresponds to $Q$ values near 1. Expanding $f(Q)$ near $Q = 1$, and assuming that $q_k \sim Dk^{-\gamma}$ as $k \to \infty$ with $2 < \gamma < 3$ yields [2]

$$f(Q) \sim 1 - f'(1)(1 - Q) + D\Gamma(1 - \gamma)(1 - Q)^{\gamma - 1} \quad \text{as } Q \to 1^- \tag{46}$$

where $\Gamma$ is the Gamma function. If the offspring distribution has a finite second moment then Supplementary Equation (46) is replaced by

$$f(Q) \sim 1 - f'(1)(1 - Q) + \frac{f''(1)}{2}(1 - Q)^2 \quad \text{as } Q \to 1^-. \tag{47}$$

In the critical case $f'(1) = 1$ and substituting from Supplementary Equation (46) into Supplementary Equation (45) gives

$$\int \frac{dQ}{D\Gamma(1 - \gamma)(1 - Q)^{\gamma - 1}} = \int dt$$

which integrates to

$$1 - Q \sim C_1 t^{-\frac{1}{\gamma - 2}} \quad \text{as } t \to \infty, \tag{48}$$

where $C_1 = [D(\gamma - 2)\Gamma(1 - \gamma)]^{-\frac{1}{\gamma - 2}}$. (If $f''(1)$ is finite, then Supplementary Equation (48) still holds with $\gamma$ replaced by 3 and $C_1$ replaced by $2/f''(1)$.)

Differentiating Supplementary Equation (46) (or Supplementary Equation (47)) and substituting for $Q(t)$ from Supplementary Equation (48) leads to

$$f'(Q(t)) \sim 1 - C_2 t^{-1} \quad \text{as } t \to \infty \tag{49}$$

and hence Supplementary Equation (21) yields the asymptotic avalanche shape, in the limit $T \to \infty$ and $T - t \to \infty$, as

$$A(t) \sim C_3 \, T^{\frac{1}{\gamma-2}} \frac{t}{T} \left(1 - \frac{t}{T}\right)^{\frac{1}{\gamma-2}}, \tag{50}$$

where $C_2$ and $C_3 = (\gamma - 1)/C_1$ are independent of $t$ and $T$, and this formula holds also for finite $f''(1)$ if $\gamma$ is replaced by 3. We note that the asymptotic avalanche shape is parabolic for $\gamma = 3$ (i.e., if $f''(1)$ is finite), but its peak is at $t/T = (\gamma - 2)/(\gamma - 1) < 1/2$ for $\gamma$ values between 2 and 3.

Similarly, the asymptotic behaviour of the average non-terminating avalanche shape at criticality is determined from Supplementary Equation (38) to be (in the limit $T \to \infty$ and $T - t \to \infty$):

$$A_{NT}(t) \sim \begin{cases} C_4 \, t \left(2 - \frac{t}{T}\right) & \text{if } f''(1) \text{ is finite,} \\ \\ C_4 \, T^{\frac{1}{\gamma-2}} \left(1 - \left(1 - \frac{t}{T}\right)^{\frac{\gamma-1}{\gamma-2}}\right) & \text{if } q_k \sim k^{-\gamma} \text{ as } k \to \infty, \text{ with } 2 < \gamma < 3, \end{cases} \tag{51}$$

where $C_4$ is independent of $t$ and $T$.

The asymptotic behaviour of the variance of the avalanche shape is found by analyzing Supplementary Equation (26) in the same way, yielding

$$\text{var}\,(n(t)) \sim T^{\frac{2}{\gamma-2}} \frac{\gamma - 1}{C_1^2} \frac{t}{T} \left(1 - \frac{t}{T}\right)^{\frac{2}{\gamma-2}} \left(3 - \gamma + \frac{t}{T}(\gamma - 2)\right) \tag{52}$$

(with $\gamma = 3$ again recovering the finite-$f''(1)$ case). Using this formula and the asymptotics of the mean avalanche shape from Supplementary Equation (50) gives the result for the coefficient of variation of the avalanche shape in equation (10) of the main text.

## Supplementary Note 7: Effect of number of avalanches

In this Note we examine how the results of the numerical simulations are affected by the number $n_A$ of avalanches that are initiated in each simulation (and subsequently filtered by their duration $T$) in order to calculate the average avalanche shape and the other characteristic temporal shapes. Specifically, we focus on the neuronal dynamics example of Figure 6 of the main text. As described in the Methods section, the results in each panel of Fig. 6 are obtained by repeating 24 replica experiments, each of which calculates the temporal profiles of $n_A = 10^7$ avalanches. The average avalanche shape and the other temporal shapes of interest are calculated in each experiment, and the plots in Fig. 6 show the average of these results over the 24 replica experiments, with error bars denoting the standard deviations of the measure over the set of 24 replicas.

In Supplementary Figure 3 we perform similar experiments on the same networks as in Fig. 6, but we use an order of magnitude fewer avalanches in each experiment when calculating the temporal profiles, i.e., $n_A = 10^6$. Similarly, in Supplementary Figure 4 we reduce the number of avalanches simulated by another factor of 10, to $n_A = 10^5$, and in Supplementary Figure 5, the value of $n_A$ is decimated again, to $n_A = 10^4$. Comparing these figures to the original Figure 6, it is clear that using fewer avalanches leads to increased fluctuations from experiment to experiment, with consequent increases in error bars. Nevertheless, the main qualitative features that we highlight, such as the left skew of the average avalanche shape in the left column panels (corresponding to power-law networks), remain detectable even at lower values of $n_A$. Recall also that the set of

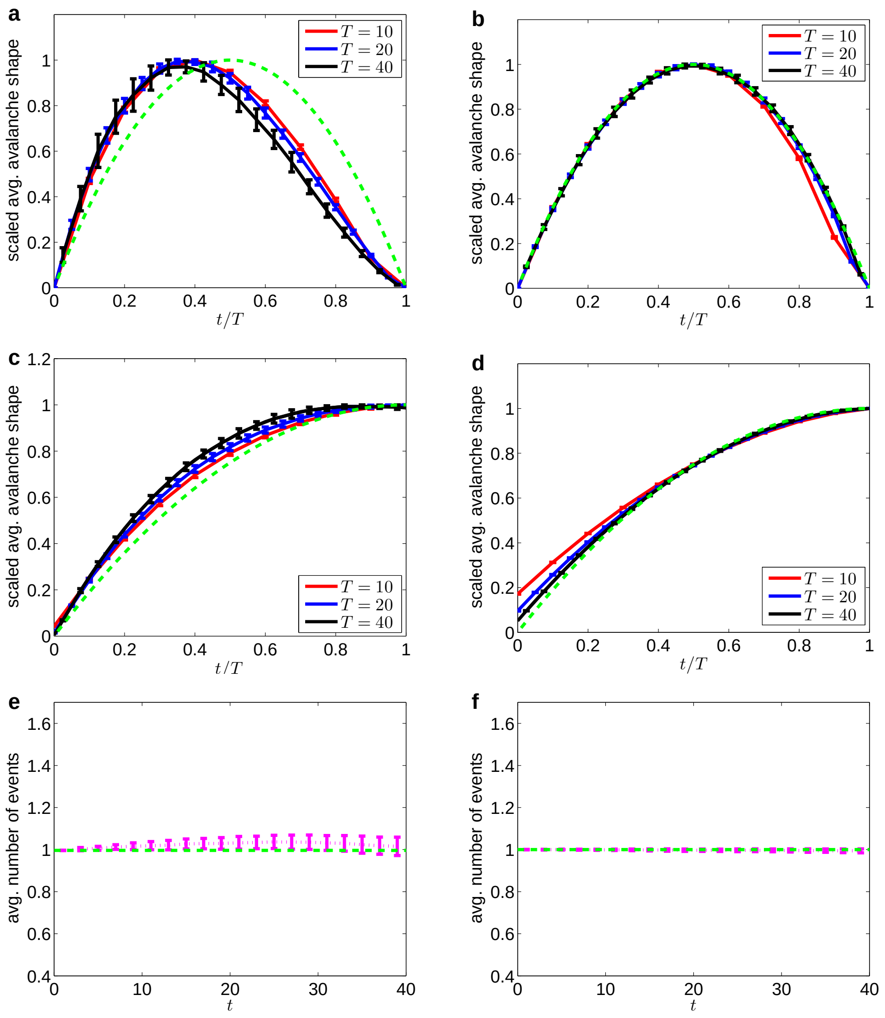


Supplementary Figure 3: | **Effect of number of avalanches: large ensemble.** As Figure 6 of the main text, but using $n_A = 10^6$ avalanches in each experiment.

avalanches that have not terminated by time $T$ is typically much larger than the set of avalanches that terminate precisely at time $T$, so we remark that the new temporal characteristics we suggest in the main text, such as the average non-terminating avalanche shape, may be more suitable for experimentalists testing for criticality than the traditional average avalanche shape. The left column panels of Supplementary Figure 5 give some evidence in support of this claim: in this case, using a relatively small data set, we find no avalanches that terminate precisely at $T = 40$ and so the black curve is absent in panel (a) of Supplementary Figure 5. However, the corresponding curve for the average non-terminating avalanche shape remains well-defined at $T = 40$, see panel (c). Thus, the use of the new characteristic temporal shape, and its comparison with the analytical predictions in Supplementary Notes 4 and 6—especially the asymptotic result of Supplementary Equation (51)—can prove useful to experimentalists seeking to quantify whether a system is in a critical state.

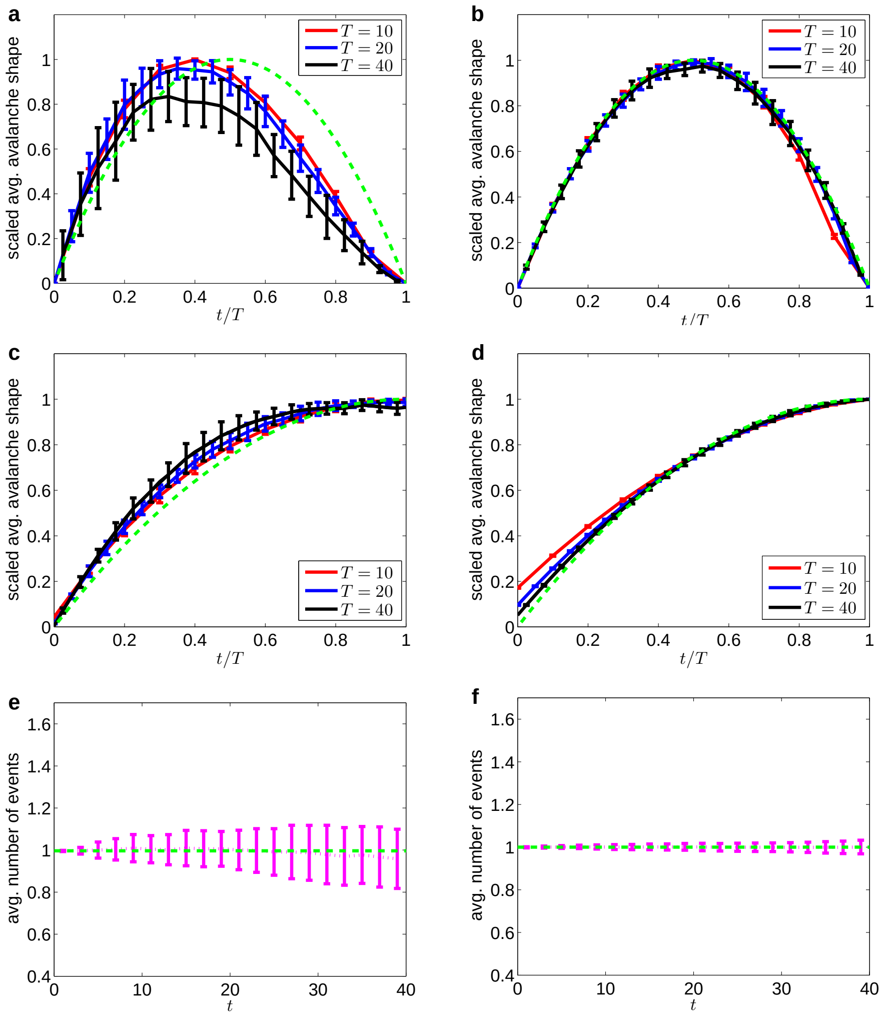


Supplementary Figure 4: | **Effect of number of avalanches: medium ensemble.** As Figure 6 of the main text, but using $n_A = 10^5$ avalanches in each experiment.

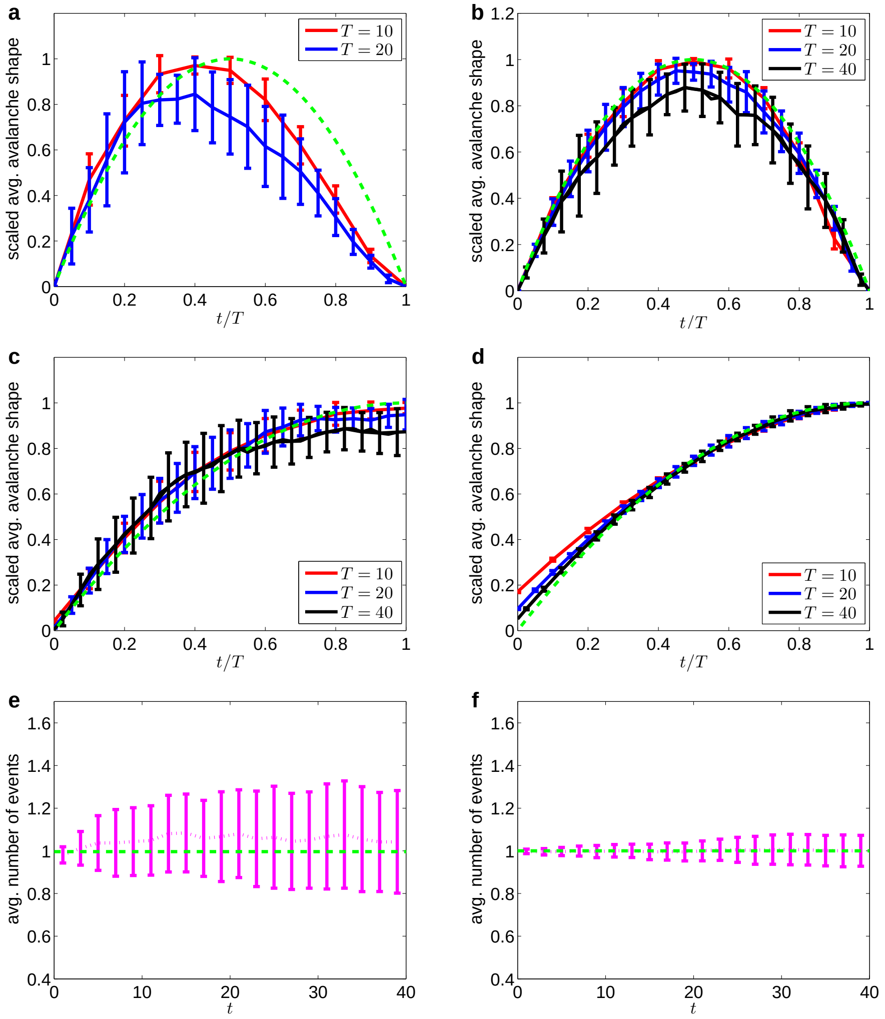


Supplementary Figure 5: | **Effect of number of avalanches: small ensemble.** As Figure 6 of the main text, but using $n_A = 10^4$ avalanches in each experiment.

## Supplementary Note 8: Further information on Figure 8

Figure 8 of the main text shows results of simulations of the meme propagation model of [3] on the empirical Twitter network of [4, 5]. Despite the fact that the empirical network has many structural features that invalidate the assumptions of our mathematical derivation (e.g., reciprocal links, clustering, degree correlations), the results of Fig. 8 appear to obey the qualitative predictions of our theory, so demonstrating that the theory's usefulness extends beyond the regime of strict assumptions required for its derivation. However, a possible counterargument to this claim could point to the structural complexity of the empirical network and hypothesize that mesoscopic or macroscopic structural features (e.g., clustering, communities) may play a more important role in determining the temporal profiles of the cascade than the degree distribution does. In this Note we therefore explore (and refute) this hypothesis, using suitably rewired versions of the empirical network [6].

For the panels in the right-hand column of Fig. 8 (panels (b), (d) and (f)), we use a directed Erdős-Rényi network with the same mean degree as the empirical network but with all other structural characteristics randomised. We create this network by simply taking each directed link of the original network and reassigning both its end nodes to be randomly-chosen nodes. This rewired network is used in Fig. 8 to show that symmetric avalanche shapes are obtained when the degree distribution does not have fat tails, as expected from the theory.

In Supplementary Figure 6 we show simulation results for a differently rewired version of the original empirical network. The rewiring algorithm used here (called "$p_{jk}$-rewiring" in [3]) preserves the degree distribution of the original network, but removes (or drastically reduces) clustering, reciprocal links, and any macro-scale structure (similar to the rewiring algorithm used in [6]). The algorithm begins by severing all links of the original network, but with each node retaining its number $j$ of "in-stubs" and $k$ of "out-stubs" that represent the endpoints of the deleted original edges. We then randomly select one out-stub and one in-stub from the entire set of stubs and create a new edge joining the selected nodes. By repeating this procedure, each time selecting from the set of unused out-stubs and in-stubs, we create a network with precisely the same $(j, k)$ distribution as the original network, but with randomised meso- and macro-scopic structure: for example, the fraction of reciprocal links is reduced from 48% in the original network to 1% in the rewired network. The panels of Supplementary Figure 6 show the results of $n_A = 1.4 \times 10^6$ avalanches over $n_R = 6$ replicas on the rewired network, and should be compared with panels (a), (c) and (e) of Fig. 8, which correspond to the original empirical network. The asymmetric avalanche shapes remain clearly visible in the rewired case of Supplementary Figure 6, which is evidence that the fundamental driver of the avalanche shape asymmetry is indeed the degree distribution (as examined in our theory), while meso- and macro-scopic structural effects are of lesser importance.

## Supplementary Note 9: Example of non-Markovian dynamics

The theoretical derivation of all our analytical results assume Markovian (Poissonian) temporal dynamics. As we note in the Discussion section, extending the theory to non-Markovian cascades is a significant challenge that lies beyond the scope of the current work. However, as a preliminary test of whether our results are robust to violations of the Markov assumption, in this Note we describe the results of numerical simulations of a non-Markovian extension of the meme-propagation model of [3].

The original model of [3] is described in terms of discrete (but small) time steps of $\Delta t = 1/N$, where $N$ is the number of nodes in the network. For large $N$ (we use $N = 10^5$ nodes), the discrete-

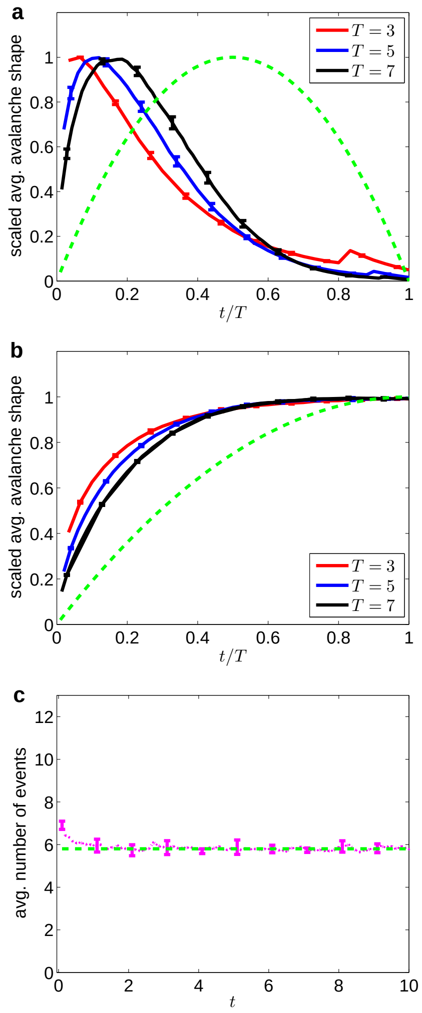


Supplementary Figure 6: | **Cascades on rewired Twitter network.** As Figure 8 of the main text, but for simulations on a $p_{jk}$-rewired version of the empirical Twitter network, which retains the degree distribution of the original network but otherwise randomizes the structure. Note that the qualitative features seen in the left column of Fig. 8 remain apparent here, which is evidence that these features are principally controlled by the degree distribution of the network (as in our theory), rather than being caused by meso- or macro-scopic structure in the empirical network. See Methods (in main text) for definition of error bars.

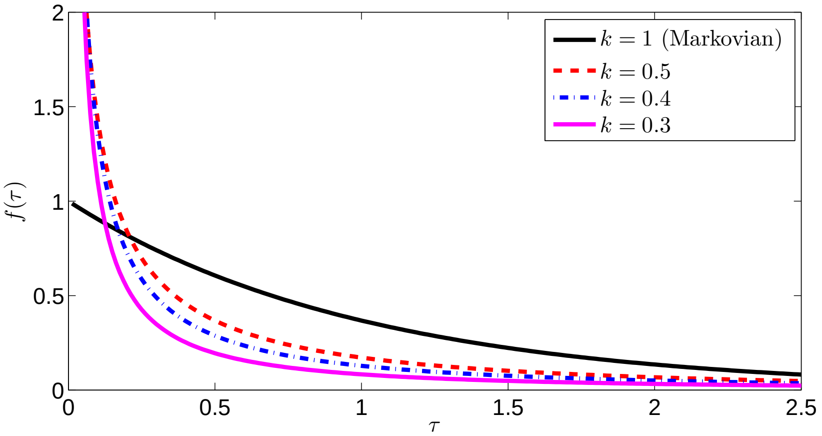


Supplementary Figure 7: | **Distributions of inter-event times.** The unit-mean Weibull probability density function $f(\tau) = \frac{k}{\lambda}\left(\frac{\tau}{\lambda}\right)^{k-1} e^{-(\tau/\lambda)^k}$ with shape parameter $k$ and scale parameter $\lambda = 1/\Gamma\left(1 + 1/k\right)$, for the values of $k$ used in the numerical simulations of Supplementary Figures 8 to 10.

time dynamics is a good approximation to Markovian continuous-time updating, where each node waits an exponentially-distributed time $\tau_n$ after its last tweet before it tweets again. The time scales of the model are chosen so that the mean inter-event time (average of $\tau_n$) is 1.

For the non-Markovian generalization of the model, simulations are run in continuous time and each user becomes active (sends a tweet) at times that are separated by intervals $\tau_n$ drawn from a unit-mean Weibull distribution with shape parameter $k$. To ensure the dynamics are stationary from the beginning, the time to the first tweeting of each node is drawn from the corresponding residual waiting time distribution [7]. For $k = 1$, the inter-event time distribution is exponential, and we recover the results of the original Markovian model shown in Figure 5 of the main text. In Supplementary Figures 8 through 10 we successively decrease the parameter $k$ to investigate the impact of increasingly non-Markovian dynamics, where short inter-event times become more probable [8], see Supplementary Figure 7. In each case we use $n_A = 1.9 \times 10^5$ avalanches in $n_R = 6$ replicas; the innovation parameter is $\mu = 0$. Supplementary Figure 8 shows results for $k = 0.5$, which are qualitatively similar to the $k = 1$ Markovian case of Fig. 5, except for some early-time effects (compare, in particular, the panels (e) and (f) in Fig. 5 and in Supplementary Figure 8). Our main result, that the symmetry of the avalanche shape functions depends on whether or not the offspring distribution has a power-law tail, certainly seems robust to the non-Markovian effects (compare panels (a) and (b)). We therefore continue towards increasingly non-Markovian dynamics in Supplementary Figures 9 and 10, where the Weibull shape parameter is further reduced to, respectively, $k = 0.4$ and $k = 0.3$. The early-time effect is more extreme in these cases, which also impacts upon the early-time avalanche shapes. Nevertheless, the qualitative results do seem to be robust for at least moderately non-Markovian dynamics, indicating that the domain of possible applicability of our theoretical results is not limited solely to Markovian dynamics. Of course, a rigorous analysis of non-Markovian effects requires an extensive theoretical analysis; we hope the present paper will form a basis for such future work.

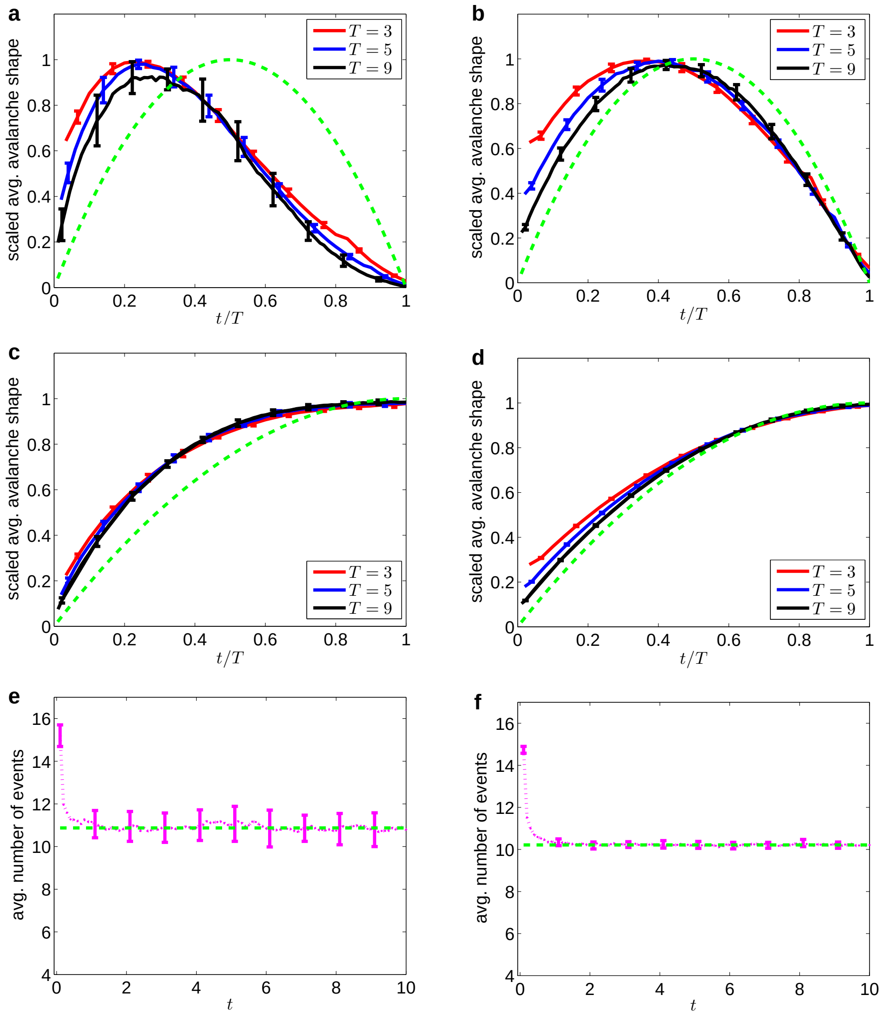


Supplementary Figure 8: | **Non-Markovian dyanamics, $k = 0.5$.** Non-Markovian generalization of the meme propagation model; compare to the Markovian case in Fig. 5 of the main text. Here the shape parameter of the Weibull distribution of inter-event times is $k = 0.5$. Note that panels (a) through (d) are quite similar to the corresponding panels of Fig. 5; panels (e) and (f) clearly show the non-Markovian effects on the early time evolution of the avalanches, but the long-time behaviour is again similar to that of Fig. 5. See Methods (in main text) for definition of error bars.

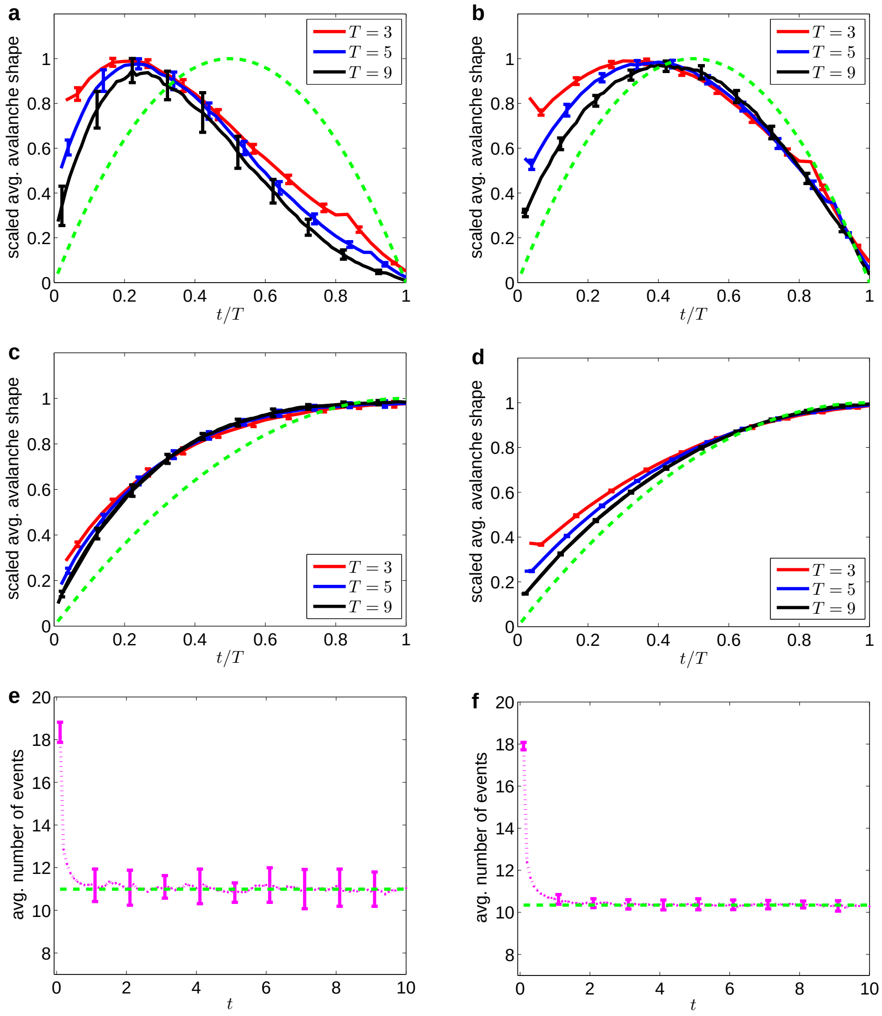


Supplementary Figure 9: | **Non-Markovian dynamics,** $k = 0.4$**.** As Supplementary Figure 8, but for Weibull shape parameter $k = 0.4$. Note the increased vertical scale in panels (e) and (f).

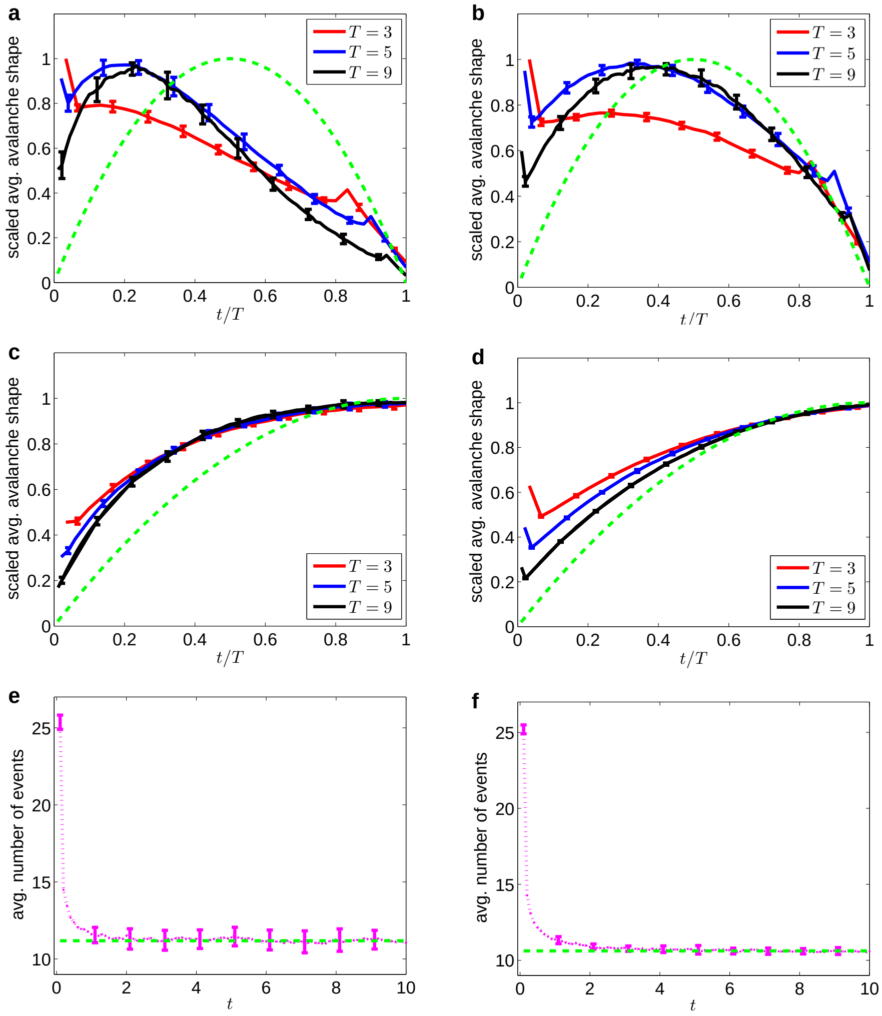


Supplementary Figure 10: | **Non-Markovian dynamics,** $k = 0.3$**.** As Supplementary Figure 8, but for Weibull shape parameter $k = 0.3$. Note the increased vertical scale in panels (e) and (f).

# Supplementary References


[1] Athreya, K. B. & Ney, P. E. *Branching Processes* (Springer Science & Business Media, 2012).

[2] Wilf, H. S. *generatingfunctionology* (Elsevier, 2013).

[3] Gleeson, J. P., Ward, J. A., O'Sullivan, K. P. & Lee, W. T. Competition-induced criticality in a model of meme popularity. *Phys. Rev. Lett.* **112**, 048701 (2014).

[4] SNAP Network datasts. `http://snap.stanford.edu/data/egonets-Twitter.html`.

[5] McAuley, J. J. & Leskovec, J. Learning to discover social circles in ego networks. In *Proc. Neural Information Processing Systems Conf. 2012*, 548–556 (2012).

[6] Karsai, M. *et al.* Small but slow world: How network topology and burstiness slow down spreading. *Phys. Rev. E* **83**, 025102 (2011).

[7] Jo, H.-H. *et al.* Analytically solvable model of spreading dynamics with non-Poissonian processes. *Phys. Rev. X* **4**, 011041 (2014).

[8] Gleeson, J. P. *et al.* Effects of network structure, competition and memory time on social spreading phenomena. *Phys. Rev. X* **6**, 021019 (2016).
